# Citrus Peel Xanthophylls Orchestrate Lipid–Glucose–Inflammation Axis Reprogramming in Cardiometabolic Syndrome: Integrative In Silico and In Vivo Evidence

**DOI:** 10.1002/mnfr.70576

**Published:** 2026-08-04

**Authors:** Muhammad Reva Aditya, Muhammad Yusuf, Kanandya Kizzandy, Rizki Hari Mulia, Agnes Vianne, Athaya Rahmanardi Muhammad, Davina Shafa Aviantiputri, Adha Fauzi Hendrawan, Ade Meidian Ambari, Dante Saksono Harbuwono, Antonello Santini, Fahrul Nurkolis

**Affiliations:** ^1^ Faculty of Medicine Universitas Brawijaya Malang East Java Indonesia; ^2^ Department of Medicine Faculty of Medicine Public Health, and Nursing Universitas Gadjah Mada Yogyakarta Indonesia; ^3^ Division of Endocrinology, Metabolism, and Diabetes Department of Internal Medicine Faculty of Medicine Universitas Indonesia Dr. Cipto Mangunkusumo National Referral Hospital Jakarta Indonesia; ^4^ Department of Cardiology and Vascular Medicine Faculty of Medicine Universitas Indonesia—National Cardiovascular Center Harapan Kita Jakarta Indonesia; ^5^ Department of Pharmacy University of Napoli Federico II Napoli Italy; ^6^ State Islamic University of Sunan Kalijaga (UIN Sunan Kalijaga) Yogyakarta Indonesia; ^7^ Medical Research Center of Indonesia Surabaya East Java Indonesia; ^8^ Master of Basic Medical Science Faculty of Medicine Universitas Airlangga Surabaya East Java Indonesia; ^9^ Fahrul Institute for Innovation and Research (FIIR) Yogyakarta Indonesia

**Keywords:** AMPK, cardiometabolic syndrome, citrus peel, functional food, inflammation, metabolomics, network pharmacology, PPAR‐*γ*, SREBP‐1c, xanthophylls

## Abstract

Cardiometabolic syndrome (CMS) involves interconnected dysregulation of lipid, glucose, and inflammatory pathways. Citrus peel, an agro‐industrial byproduct, is rich in xanthophylls with potential multitarget effects, yet their integrated mechanisms remain unclear. This study aimed to investigate the mechanistic basis and therapeutic efficacy of a citrus peel xanthophyll fraction (CPXF) in modulating the lipid–glucose–inflammation axis through integrated in silico and in vivo approaches. CPXF was characterized via UHPLC–HRMS/MS. Network pharmacology and molecular docking identified target interactions. In vivo validation was conducted in high‐fat diet plus fructose‐induced CMS rats, evaluating metabolic, inflammatory, oxidative, and gene expression parameters. *β*‐cryptoxanthin, violaxanthin, and lutein were dominant compounds. Key targets included PPARG, AMPK, SREBP1, and MMP9, with strong binding affinities (Δ*G* up to −10.3 kcal/mol). CPXF significantly improved glucose homeostasis, insulin resistance, lipid profile, and OGTT outcomes. It reduced TNF‐*α*, IL‐6, and CRP, attenuated oxidative stress (↓MDA; ↑SOD, CAT, GSH), and restored hepatic function. Gene expression analysis showed upregulation of Ampk and Pparg and suppression of Srebp1c. CPXF exerts synergistic, multitarget effects across the lipid–glucose–inflammation axis via AMPK activation, PPAR‐*γ* modulation, and SREBP‐1c suppression, supporting its potential as a food‐derived strategy for CMS management.

## Graphical abstract

Central obesity and dyslipidaemia converge to impair insulin signalling and trigger chronic inflammation, oxidative stress, and mitochondrial dysfunction. This integrated metabolic disruption drives lipid imbalance, apoptosis, and persistent hyperglycaemia, ultimately accelerating the progression of type 2 diabetes and cardiovascular disease.

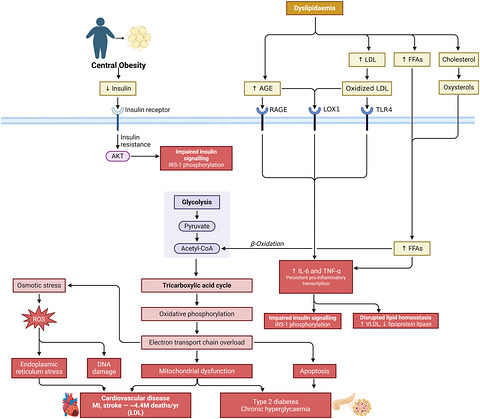

## 1 Introduction

Cardiometabolic syndrome (CMS) is a multifaceted metabolic disorder characterized by the co‐occurrence of central obesity, dyslipidemia, hyperglycemia, and chronic low‐grade inflammation [1]. CMS also independently linked with the elevated risk of cardiovascular disease (CVD) and type 2 diabetes mellitus (T2DM) [2, 3]. According to the Global Burden of Disease Study 2019, elevated low‐density lipoprotein (LDL) cholesterol contributed to approximately 4.4 million deaths globally [4]. Previous studies have also shown that CMS drives persistent transcription of pro‐inflammatory mediators, particularly interleukin‐6 (IL‐6) and tumor necrosis factor‐alpha (TNF‐*α*), which in turn impair insulin receptor signaling and disrupt lipid homeostasis [5, 6]. Although pharmacological agents such as statins, fibrates, and thiazolidinediones remain the clinical standard of care, their long‐term utility is constrained by the side effects [7, 8]. These limitations have intensified interest in naturally derived, multitarget bioactive compounds capable of modulating the cardiometabolic pathology.

Citrus peel, a major byproduct of global fruit processing industries, constitutes approximately 50% of total fruit mass and is routinely discarded despite its concentration of phenolic compounds, carotenoids, essential oils, and vitamins [9]. Among the phytochemical classes abundant in citrus peel, xanthophylls oxygenated carotenoids remain mechanistically underexplored relative to flavonoids, despite mounting evidence of biological potency and favorable bioavailability [10, 11]. The predominant citrus derived xanthophylls, *β*‐cryptoxanthin (BCX), lutein, and zeaxanthin, are concentrated in tangerines and sweet oranges, with BCX displaying markedly higher bioavailability than most carotenoids [12]. Dietary intake of these compounds is associated with reduced incidence of CVD and age‐related chronic conditions [13].

These xanthophylls exert coordinated effects across the lipid–glucose–inflammation axis through complementary but mechanistically distinct pathways. In animal models, lutein and zeaxanthin reduce oxidized LDL, circulating inflammatory cytokines, blood pressure, and lipid parameters while improving glycemic control [14, 15]. Clinically, plasma lutein and zeaxanthin concentrations correlate inversely with IL‐6 in coronary artery disease patients, and lutein exposed peripheral blood mononuclear cells display markedly attenuated inflammatory responses [16]. At the lipid metabolism interface, BCX reduces body fat and transcriptionally upregulates energy metabolism [17]. Despite this breadth of activity, the structural determinants governing xanthophyll interactions with key metabolic enzymes remain poorly defined for citrus‐derived species.

Closing the gap between epidemiological and mechanistic requires an evidence‐tiered framework. In silico approaches enable rational prioritization of phytochemical target interactions before animal experimentation. In silico methodologies enable rational, resource‐efficient identification of phytochemical target interactions prior to animal experimentation. While systematic evidence confirms that higher circulating lutein is associated with reduced metabolic syndrome and CVD risk, the specific molecular targets and dose–response relationships governing xanthophyll bioactivity in whole‐organism cardiometabolic models remain incompletely characterized. The present study therefore applied an integrated in silico and in vivo pipeline, then the key predicted interactions were then experimentally validated in a rodent model of CMS. We hypothesized that the combined xanthophyll fraction would exert pleiotropic, potentially synergistic effects across the lipid–glucose–inflammation axis, yielding both mechanistic insight and experimental support for citrus peel xanthophylls as food‐derived candidates for evidence‐based CMS management.

## 2 Materials and Methods

### 2.1 Preparation and Enrichment of Citrus Peel Xanthophyll Fraction

Fresh citrus peels of *Citrus sinensis* were obtained from fully ripened fruits and authenticated by a qualified botanist, 1000 g of dried *C. sinensis* peel powder was extracted, yielding 118.5 g of crude extract (11.85%, w/w). Following silica gel column chromatography, 24.7 g of citrus peel xanthophyll fraction (CPXF) was obtained, corresponding to 20.8% of the crude extract and an overall yield of 2.47% based on the dried plant material. The peels were thoroughly washed with distilled water to remove surface impurities, air‐dried at controlled temperature (40 ± 2°C) to prevent degradation of thermolabile compounds, and subsequently milled into a fine powder using a laboratory grinder. The powdered material was subjected to extraction using a solvent system consisting of ethanol and hexane (1:1, v/v), which is suitable for extracting both polar and nonpolar carotenoid derivatives, particularly oxygenated carotenoids (xanthophylls). The extraction was carried out under continuous magnetic stirring for 24 h at room temperature in dark conditions to minimize photo‐oxidation.

The extract was filtered through Whatman No. 1 filter paper and concentrated under reduced pressure using a rotary evaporator at temperatures not exceeding 40°C. The crude extract was then subjected to liquid–liquid partitioning to remove polar impurities, followed by silica gel column chromatography (60–120 mesh). Fractionation was performed using a gradient elution system of hexane and acetone, with increasing polarity to selectively isolate oxygenated carotenoids. Fractions were monitored based on their characteristic yellow–orange coloration and UV–Vis absorbance spectra (*λ*max ≈ 445–475 nm). Fractions enriched in xanthophyll compounds were pooled, concentrated, and designated as the CPXF. The extract was stored in amber vials under nitrogen atmosphere at −20°C until further analysis to prevent oxidative degradation.

### 2.2 LC–MS/MS‐Based Metabolomic Profiling of CPXF

Metabolomic profiling of CPXF was performed using ultra‐high‐performance liquid chromatography coupled with high‐resolution tandem mass spectrometry (UHPLC–HRMS/MS). Chromatographic separation was achieved using a reverse‐phase C18 column (2.1 × 100 mm, 1.7 µm particle size) maintained at 40°C. The mobile phase consisted of (A) water containing 0.1% formic acid and (B) a mixture of acetonitrile and isopropanol (1:1, v/v), optimized for efficient separation of lipophilic carotenoids.

A gradient elution program was employed starting from 30% B, linearly increased to 95% B over 15 min, held for 3 min, and then returned to initial conditions for column re‐equilibration. The flow rate was maintained at 0.3 mL/min, and the injection volume was set at 5 µL. Detection was carried out using a high‐resolution mass spectrometer operated in positive electrospray ionization (ESI+) mode, which is suitable for ionization of carotenoid derivatives. Instrument parameters were optimized as follows: capillary voltage at 3.5 kV, capillary temperature at 320°C, sheath gas at 35 arbitrary units, and auxiliary gas at 10 arbitrary units. Data acquisition was performed in a full‐scan mode (*m*/*z* 100–1500), followed by data‐dependent MS/MS fragmentation for structural elucidation. Data acquisition was performed in both positive and negative electrospray ionization (ESI+/ESI−) modes to maximize metabolite coverage. However, carotenoid‐derived metabolites exhibited substantially stronger signal intensity, better ion stability, and more informative fragmentation patterns under positive ionization. Therefore, only the positive ESI data were used for metabolite annotation and subsequent analyses.

Raw LC–MS/MS data were processed using Compound Discoverer (Thermo Scientific) with retention time alignment tolerance set at 0.2 min and mass accuracy tolerance of 5 ppm. Background noise and blank signals were removed to improve data quality. Compound identification was performed through accurate mass matching and comparison of MS/MS fragmentation patterns against spectral libraries including mzCloud, Human Metabolome Database (HMDB), and METLIN. Metabolites were annotated at Metabolomics Standards Initiative (MSI) level 2 (putatively annotated compounds). Relative abundance of each metabolite was calculated as a percentage of total ion intensity, allowing semi‐quantitative comparison of xanthophyll composition.

### 2.3 In Silico Network Pharmacology Analysis and Molecular Docking

#### 2.3.1 Network Pharmacology

Potential protein targets for citrus peel–derived compounds were identified using the SuperPred web‐based platform (https://prediction.charite.de/, accessed March 27, 2026) [18]. Compound structures were input as SMILES strings, and target predictions were filtered to retain only those achieving confidence scores of 80% or higher on the platform's 0%–100% scale, ensuring adequate prediction reliability.

Disease‐associated genes and proteins linked to CMS were retrieved from the Gene cards database (https://www.genecards.org/, accessed March 27, 2026) [19]. To identify common targets between the computationally predicted compound targets and known disease‐related proteins, overlap analysis was performed. The resulting intersections were visualized using Venn diagrams online tools (https://bioinformatics.psb.ugent.be/webtools/Venn/, accessed March 27, 2026).

Functional relationships between computationally predicted compound targets and disease‐related proteins were investigated through protein–protein interaction (PPI) network analysis using the STRING database (version 12, https://string‐db.org/, accessed March 27, 2026). This comprehensive platform consolidates both experimentally determined and computationally predicted protein interactions based on functional associations [20].

Network construction was limited to human proteins (*Homo sapiens*) to maintain biological relevance for the study objectives. The analysis incorporated all identified target proteins without filtering, employing a minimum interaction confidence threshold of 0.4 to establish moderate‐confidence connections within the network. The generated interaction data were subsequently exported for further computational analysis.

#### 2.3.2 Molecular Docking

Molecular docking simulations were performed using the CB‐Dock2 web server (https://cadd.labshare.cn/cb‐dock2/, accessed March 27, 2026), which employs cavity‐guided blind docking methodology incorporating binding site identification, docking calculations, and template‐based refinement to enhance predictive accuracy [21]. The platform autonomously detects potential binding sites and establishes docking parameters, including grid center coordinates and box dimensions, based on ligand properties. Generated binding conformations are evaluated and prioritized using Vina scoring functions, with comprehensive three‐dimensional interaction maps provided for analysis. Cavity identification was optimized through implementation of the CurPocket curvature‐based detection algorithm. Protein structures underwent preprocessing via the CB‐Dock2 pipeline to eliminate crystallographic water molecules, ensuring accurate ligand–protein interaction assessment.

Ligand three‐dimensional coordinates were sourced from the PubChem database, while target protein structures were obtained from the Protein Data Bank [22]. The selected protein targets comprised PRKAA1 (PDB ID: 4QFR), SREBP1c (PDB ID: 1AM9), MMP9 (PDB ID: 1GKC), PPARG (PDB ID: 2PRG), and AMPK (PDB ID: 4CFF). These targets were chosen based on their critical involvement in metabolic regulation, matrix remodeling, and cellular energy homeostasis pathways that influence various physiological processes.

### 2.4 Experimental Animals and Induction of CMS

Male Wistar rats aged 8–10 weeks with initial body weights ranging from 180 to 200 g were obtained from a certified laboratory animal facility. Animals were housed under controlled environmental conditions (temperature 22 ± 2°C, relative humidity 55%–65%, 12 h light/dark cycle) with free access to food and water. All procedures were conducted in accordance with institutional guidelines for animal care and use. All animal experimental procedures were performed in compliance with the ARRIVE (Animal Research: Reporting of In Vivo Experiments) guidelines. The study protocol was prospectively registered and received ethical approval from the Ethics Committee of the Faculty of Medicine, Brawijaya University (Approval No. 201/EC/KEPK/05/2025).

Prior to intervention, animals were acclimatized for 1 week and then randomized into 4 experimental groups (*n* = 5 per group) with comparable baseline body weights to ensure homogeneity. CMS was induced by administering a high‐fat diet (45%–60% kcal from fat) combined with fructose supplementation (10%–20% w/v in drinking water) for 6 weeks. This combined dietary model was selected to mimic both lipid overload and excessive sugar intake, thereby inducing insulin resistance, dyslipidemia, and low‐grade systemic inflammation.

### 2.5 Intervention With CPXF

Following successful induction of cardiometabolic disturbances, animals received daily oral administration of CPXF for 8 weeks. The treatment groups included a low‐dose group (50 mg/kg body weight) and a high‐dose group (100 mg/kg body weight), while the high fat diet (HFD) + fructose group received vehicle only. The normal control group was maintained on a standard diet without intervention. The extract was administered via oral gavage to ensure precise dosing.

### 2.6 Anthropometric and Biochemical Assessments

Body weight was measured weekly throughout the experimental period using a calibrated digital balance. At the end of the intervention, animals were fasted overnight (12 h) prior to sample collection. Blood samples were obtained via cardiac puncture under anesthesia and centrifuged at 3000 rpm for 15 min to separate serum.

Fasting blood glucose levels were measured using an enzymatic colorimetric assay, while serum insulin concentrations were quantified using a commercially available ELISA kit according to the manufacturer's instructions. Insulin resistance was evaluated using the Homeostatic Model Assessment of Insulin Resistance (HOMA‐IR), calculated as:

HOMA−IR=(fastingglucose×fastinginsulin)/405

Serum lipid parameters, including total cholesterol, triglycerides, LDL cholesterol, and high‐density lipoprotein cholesterol, were determined using enzymatic assay kits following standard protocols. Serum alanine aminotransferase (ALT) and aspartate aminotransferase (AST) were additionally determined using commercially available enzymatic assay kits to assess hepatic function.

### 2.7 Oral Glucose Tolerance Test (OGTT)

Glucose tolerance was assessed using an OGTT performed after overnight fasting. Animals were orally administered glucose at a dose of 2 g/kg body weight. Blood glucose levels were measured at 0, 30, 60, and 120 min post‐glucose administration using a glucometer. The area under the curve (AUC) was calculated using the trapezoidal method to quantify overall glucose excursion.

### 2.8 Assessment of Inflammatory and Oxidative Stress Biomarkers

Serum concentrations of pro‐inflammatory cytokines, including tumor TNF‐*α* and interleukin‐6 (IL‐6), as well as C‐reactive protein (CRP), were quantified using enzyme‐linked immunosorbent assay (ELISA) kits according to the manufacturer's protocols.

Liver tissues were excised, washed in ice‐cold saline, and homogenized in phosphate buffer. The homogenates were centrifuged, and the supernatant was used for analysis of oxidative stress markers. Lipid peroxidation was assessed by measuring malondialdehyde (MDA) levels, while antioxidant enzyme activities, including superoxide dismutase (SOD) and catalase (CAT), were determined spectrophotometrically. Reduced glutathione (GSH) levels were also measured. All oxidative stress parameters were normalized to total protein content to ensure comparability.

### 2.9 Gene Expression Analysis

Total RNA was extracted from liver tissues using TRIzol reagent following standard protocols. The purity and concentration of RNA were assessed spectrophotometrically. Complementary DNA (cDNA) was synthesized using a reverse transcription kit, and quantitative real‐time PCR (qPCR) was performed using SYBR Green Master Mix.

Gene expression analysis focused on key regulators of lipid metabolism, glucose homeostasis, and inflammation, including peroxisome proliferator‐activated receptor gamma (Pparg), AMP‐activated protein kinase (Ampk), sterol regulatory element‐binding protein 1c (Srebp1c), tumor necrosis factor (TNF), and interleukin‐6 (Il6). Relative gene expression levels were calculated using the 2^−^ΔΔ*Ct* method and normalized to a housekeeping gene (GAPDH).

### 2.10 Statistical Analysis

All data were expressed as mean ± standard deviation (SD). Statistical analyses were performed using one‐way analysis of variance (ANOVA) followed by Tukey's post hoc test to determine differences among groups. A *p*‐value of less than 0.05 was considered statistically significant. For tabular presentation, results were annotated using different superscript letters (a, b, c) to indicate statistically significant differences between groups within the same row, ensuring clear interpretation of group comparisons.

## 3 Results

### 3.1 Metabolomic Characterization of CPXF

LC–MS/MS‐based metabolomic profiling of the CPXF identified a total of eight carotenoid compounds, predominantly belonging to the xanthophyll class (Table 1). Among these, BCX was the most abundant compound, with a mean concentration of 185.4 mg/g and a relative percentage of 31.8%, followed by violaxanthin (132.7 mg/g; 22.8%) and lutein (96.8 mg/g; 16.6%). These three compounds represented the major carotenoid constituents of CPXF, indicating that the fraction was dominated by oxygenated carotenoids. Other carotenoids detected in CPXF included zeaxanthin (61.2 mg/g; 10.5%), antheraxanthin (42.5 mg/g; 7.3%), and neoxanthin (28.6 mg/g; 4.9%), while luteoxanthin (18.1 mg/g; 3.1%) and the apo‐10′‐carotenal derivative (9.4 mg/g; 1.6%) were present at lower levels.

**TABLE 1:** LC–MS/MS‐based metabolomic profiling of carotenoid compounds in citrus peel xanthophyll fraction (CPXF).

Compound	Class	Mean (mg/g)	SD	Relative (percent)
β‐cryptoxanthin	Xanthophyll	185.4	12.6	31.8
Violaxanthin	Xanthophyll epoxy‐carotenoid	132.7	10.4	22.8
Lutein	Xanthophyll	96.8	8.1	16.6
Zeaxanthin	Xanthophyll	61.2	5.7	10.5
Antheraxanthin	Xanthophyll	42.5	4.9	7.3
Neoxanthin	Xanthophyll	28.6	3.2	4.9
Luteoxanthin	Oxygenated carotenoid	18.1	2.3	3.1
Apo‐10'‐carotenal derivative	Apocarotenoid	9.4	1.1	1.6

### 3.2 Network Pharmacology Analysis

**FIGURE 1:**
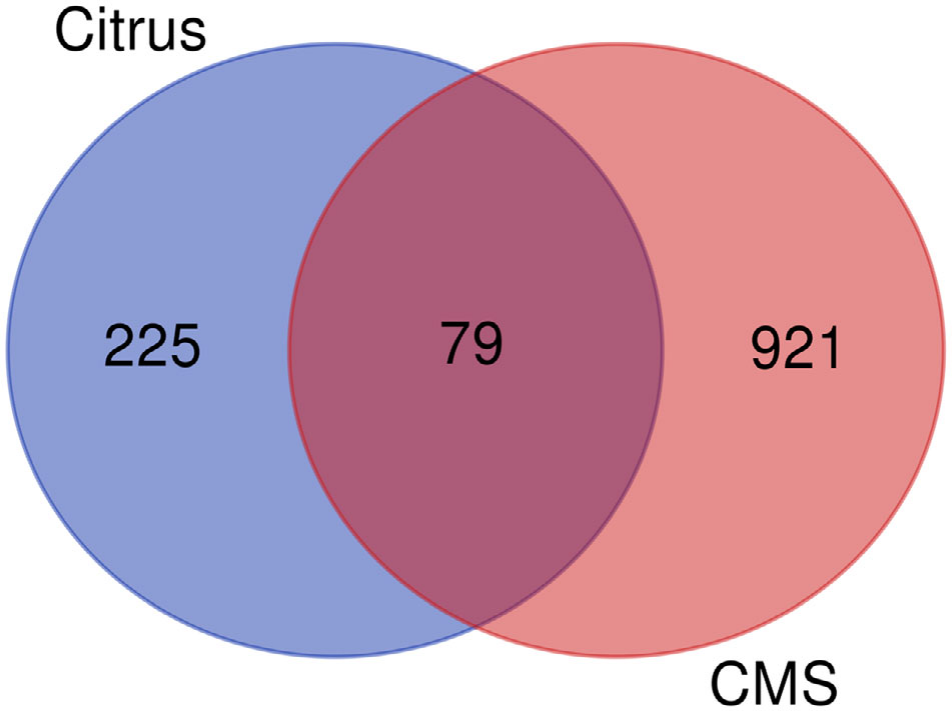
Comparative metabolomic analysis of citrus and cardiometabolic syndrome (CMS) using Venn diagram.

The comparative analysis between citrus‐derived metabolites and CMS‐related targets identified a total of 79 overlapping metabolites/targets, as illustrated in Figure 1. In addition, 225 metabolites were found to be unique to citrus, while 921 targets were specific to CMS. The presence of shared components suggests potential biological relevance of citrus‐derived compounds in modulating pathways associated with CMS. To further elucidate the biological significance of these shared metabolites and their associated targets, a protein–protein interaction (PPI) network was constructed.

**FIGURE 2:**
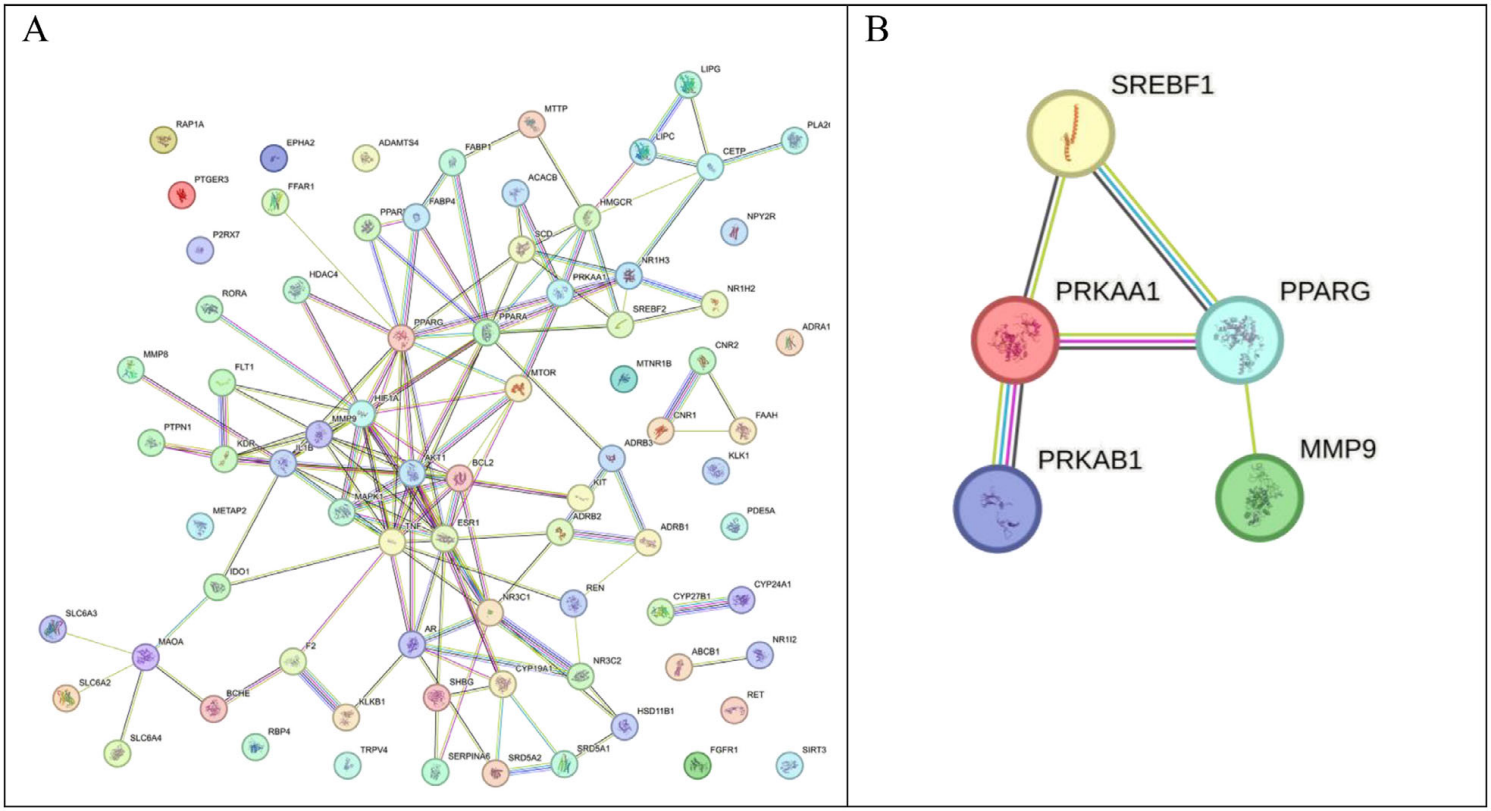
Protein–protein interaction (PPI) network of target genes and identification of key hub genes. (A) PPI network of target genes. (B) Core hub gene subnetwork.

The PPI network of the identified target genes was constructed to explore the molecular interactions associated with CPXF and CMS (Figure 2A). The network revealed a complex interaction pattern with multiple interconnected nodes, indicating strong functional relationships among the target proteins. Several genes were observed to occupy central positions within the network, suggesting their potential regulatory importance in the underlying biological processes.

Further analysis identified key hub genes, including PPARG, PRKAA1, PRKAB1, SREBF1, and MMP9, as shown in the core subnetwork (Figure 2B). These hub genes are known to be involved in lipid metabolism, energy homeostasis, and inflammatory responses, which are critical pathways in CMS. The identification of these central nodes highlights their potential role as key targets through which citrus‐derived compounds may exert therapeutic effects on CMS.

### 3.3 Molecular Docking Analysis

Molecular docking analysis was performed to evaluate the binding affinities of CPXF‐derived carotenoids against key CMS‐related target proteins, including PRKAA1, SREBP1c, MMP9, PPARG, and AMPK (Table 2). Overall, the carotenoid compounds exhibited strong binding interactions, with Δ*G* values generally lower than those of the control drugs (simvastatin and metformin), indicating favorable binding potential. Among the tested compounds, luteoxanthin showed the strongest binding affinity toward MMP9 (−10.3 kcal/mol), followed by zeaxanthin toward PPARG (−9.7 kcal/mol), and BCX toward PRKAA1 (−9.4 kcal/mol).

**TABLE 2:** Molecular docking binding affinity (Δ*G*, kcal/mol) of CPXF‐derived carotenoids against key cardiometabolic syndrome (CMS) target proteins.

	Ligands Free Binding Energy or Δ*G* (kcal/mol)				
	CMS				
Compounds	PRKAA1 (PDB ID: 4QFR)	SREBP1c (PDB ID: 1AM9)	MMP9 (PDB ID: 1GKC)	PPARG (PDB ID: 2PRG)	AMPK (PDB ID: 4CFF)
Simvastatin/Control	−8.4	−6.3	−7.0	−8.5	−7.9
Metformin/Control	−5.2	−4.1	−5.2	−5.7	−5.0
β‐cryptoxanthin	−9.4	−7.5	−9.3	−8.4	−8.3
Violaxanthin	−9.1	−8.3	−9.6	−9.4	−7.9
Lutein	−8.3	−7.2	−8.7	−9.0	−8.0
Zeaxanthin	−9.3	−7.5	−9.2	−9.7	−8.2
Antheraxanthin	−8.7	−7.3	−9.3	−8.6	−8.3
Neoxanthin	−9.3	−7.8	−9.5	−8.9	−8.4
Luteoxanthin	−9.6	−7.4	−10.3	−9.3	−8.1
Apo‐10'‐carotenal derivative	−7.8	−6.4	−9.1	−8.3	−7.4

In comparison to the controls, most carotenoids demonstrated comparable or stronger binding affinities across multiple targets. Notably, violaxanthin and neoxanthin exhibited consistently strong interactions with MMP9 and PRKAA1, while lutein and antheraxanthin also showed stable binding across all targets. These findings suggest that CPXF‐derived carotenoids possess significant potential to interact with key proteins involved in lipid metabolism and energy regulation, supporting their possible role in the management of CMS.

### 3.4 Initial Body Weight Characteristics of Among Experimental Groups

The baseline body weight of all experimental groups is presented in Table 3. The mean body weight ranged from 200.86 to 202.40 g, with the HFD + fructose group showing the highest mean value (202.40 ± 3.02 g), while the low‐dose group had the lowest (200.86 ± 1.91 g). The high‐dose and normal groups demonstrated comparable baseline weights of 201.46 ± 1.48 g and 201.64 ± 2.86 g, respectively. Statistical analysis indicated no significant differences in baseline body weight among the groups (*p* > 0.05).

**TABLE 3:** Baseline body weight of experimental groups.

Group	Mean (SD)
HFD + fructose	202.40 ± 3.02^a^
High dose	201.46 ± 1.48^a^
Low dose	200.86 ± 1.91^a^
Normal	201.64 ± 2.86^a^

*Note*: Different superscript letters within the same row indicate statistically significant differences among groups (one‐way ANOVA).

### 3.5 Effect of Citrus Peel Xanthophylls on Glycemic Control and Lipid Profile

The HFD + fructose group showed clear metabolic disturbances compared with the normal group, as reflected by higher glucose, insulin, HOMA‐IR, and OGTT‐AUC values (Table 4). Fasting glucose increased from 100.49 ± 8.04 mg/dL in the normal group to 161.59 ± 12.86 mg/dL in the HFD + fructose group, while insulin increased from 0.76 ± 0.11 ng/mL to 1.96 ± 0.15 ng/mL. Similarly, HOMA‐IR rose markedly from 3.94 ± 0.53 to 13.61 ± 1.10, indicating substantial insulin resistance. OGTT‐AUC was also elevated in the HFD + fructose group, confirming impaired glucose tolerance. These findings indicate that HFD + fructose induction successfully produced a cardiometabolic condition characterized by hyperglycemia, hyperinsulinemia, and insulin resistance.

**TABLE 4:** Effects of citrus peel xanthophylls on glucose homeostasis and lipid profile in HFD + fructose‐induced cardiometabolic syndrome.

Parameter	Normal	HFD + Fructose	Low dose	High dose
Glucose (mg/dL)	100.49 ± 8.04^a^	161.59 ± 12.86^d^	135.23 ± 12.76^c^	108.69 ± 6.71^b^
Insulin (ng/mL)	0.76 ± 0.11^a^	1.96 ± 0.15^d^	1.40 ± 0.09^c^	0.93 ± 0.10^b^
HOMA‐IR	3.94 ± 0.53^a^	13.61 ± 1.10^d^	9.14 ± 0.57^c^	5.44 ± 0.52^b^
OGTT‐AUC (mg·min/dL)	13 972.95 ± 599.51^a^	25 135.17 ± 267.98^d^	20 930.49 ± 289.62^c^	17 289.12 ± 323.35^b^
TC (mg/dL)	79.56 ± 10.00^a^	145.70 ± 10.80^d^	117.88 ± 11.68^c^	101.94 ± 9.24^b^
TG (mg/dL)	71.91 ± 3.49^a^	144.85 ± 7.29^d^	108.75 ± 9.16^c^	88.47 ± 2.13^b^
LDL (mg/dL)	23.79 ± 2.77^a^	72.60 ± 2.40^d^	47.23 ± 2.58^c^	34.36 ± 4.22^b^
HDL (mg/dL)	51.81 ± 4.31^a^	30.60 ± 3.12^d^	37.29 ± 4.78^c^	43.56 ± 4.53^b^

Data are expressed as mean ± SD (*n* = 5 per group). Different superscript letters within the same row indicate statistically significant differences among groups (one‐way ANOVA followed by Tukey's post hoc test, *p* < 0.05).

Treatment with citrus peel xanthophylls improved these abnormalities in a dose‐dependent manner. Both low‐ and high‐dose groups showed lower glucose, insulin, HOMA‐IR, and OGTT‐AUC values than the HFD + fructose group, with the high‐dose group showing values closer to normal. A similar trend was observed in lipid parameters. The HFD + fructose group had significantly increased TC, TG, and LDL levels, along with decreased HDL levels, compared with the normal group. Administration of citrus peel xanthophylls reduced TC, TG, and LDL while increasing HDL, with the high dose producing a greater effect than the low dose. Overall, these results suggest that citrus peel xanthophylls ameliorated glycemic impairment, insulin resistance, and dyslipidemia in HFD + fructose‐induced rats.

### 3.6 Effect of CPXF Treatment on Body Weight, Visceral Fat, and Liver Weight

**FIGURE 3:**
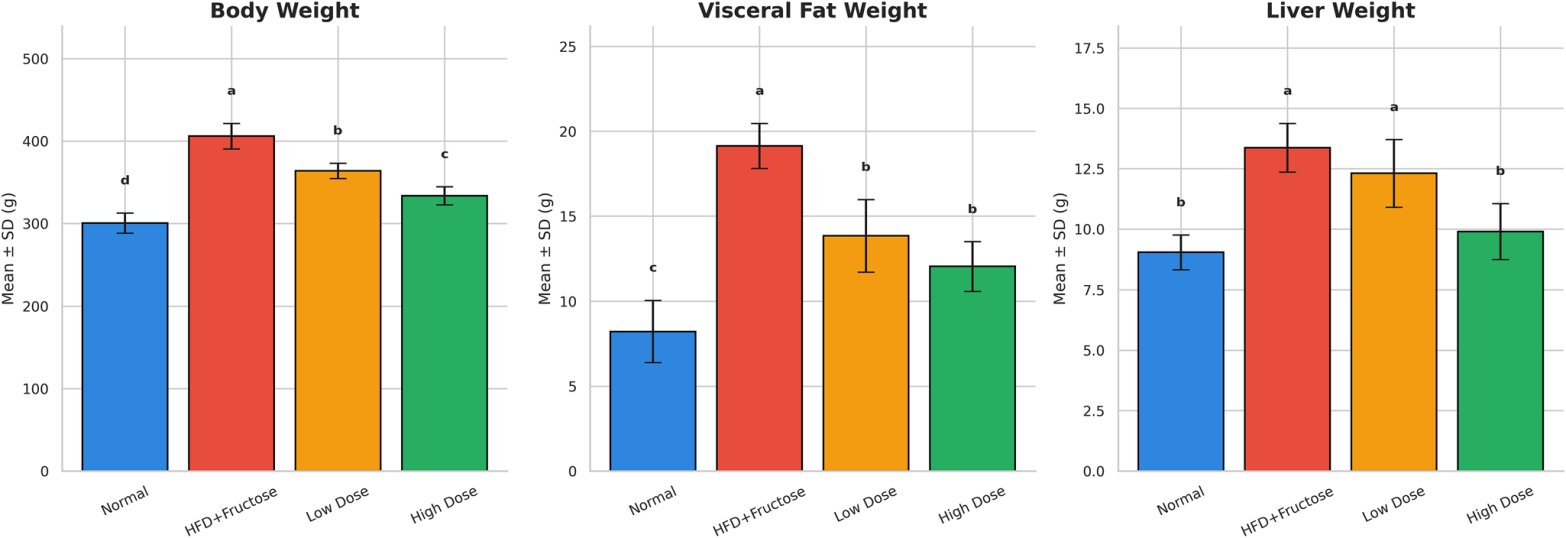
Effect of CPXF treatment on body weight, visceral fat, and liver weight in HFD‐induced rats. Data are expressed as mean ± SD (*n* = 5 per group). Statistical differences were analyzed by one‐way ANOVA followed by Tukey's post hoc test. Different superscript letters or letters above the bars indicate significant differences among groups at *p* < 0.05.

The HFD + fructose group showed a marked increase in body weight, visceral fat weight, and liver weight compared with the normal group (Figure 3). Body weight increased from 300.46 ± 12.21 g in the normal group to 405.94 ± 15.59 g in the HFD + fructose group. Similarly, visceral fat weight increased from 8.22 ± 1.83 g to 19.14 ± 1.33 g, while liver weight increased from 9.04 ± 0.72 g to 13.37 ± 1.01 g. These findings indicate that HFD + fructose induction successfully promoted obesity‐related changes, including increased adiposity and liver enlargement. Based on the superscript annotations, all groups differed significantly for body weight, whereas visceral fat and liver weight also showed significant differences among most groups at *p* < 0.05.

Treatment with CPXF attenuated these alterations in both dose groups. In the low‐dose group, body weight, visceral fat weight, and liver weight decreased to 363.90 ± 9.29 g, 13.84 ± 2.14 g, and 12.31 ± 1.40 g, respectively, compared with the HFD + fructose group. A greater improvement was observed in the high‐dose group, with body weight reduced to 333.64 ± 11.06 g, visceral fat weight to 12.04 ± 1.46 g, and liver weight to 9.90 ± 1.16 g. The high dose brought liver weight close to the normal group and produced a stronger reduction in body weight and visceral fat than the low dose.

### 3.7 Effect of CPXF Treatment on Pro‐Inflammatory Markers

**FIGURE 4:**
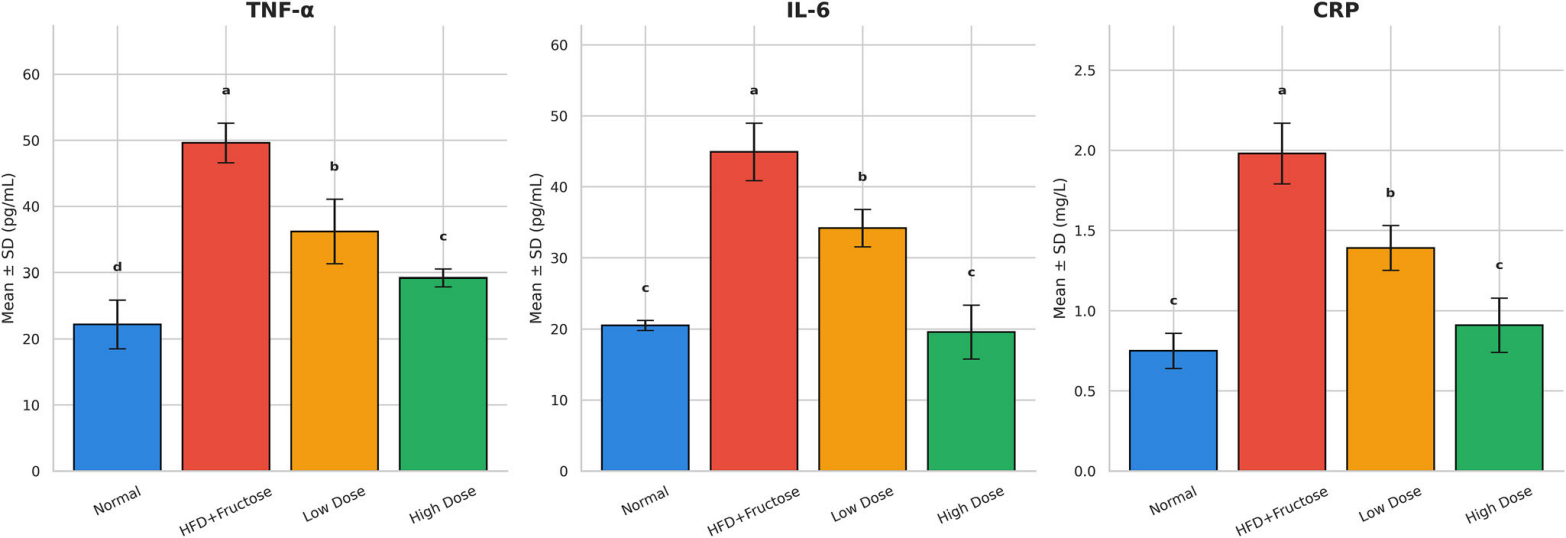
Effect of CPXF treatment on pro‐inflammatory markers (TNF‐*α*, IL‐6, and CRP) in HFD‐induced rats. Data are expressed as mean ± SD (*n* = 5 per group). Statistical differences were analyzed by one‐way ANOVA followed by Tukey's post hoc test. Different superscript letters or letters above the bars indicate significant differences among groups at *p* < 0.05.

The HFD + fructose group showed a marked increase in pro‐inflammatory markers compared with the normal group, indicating the development of a systemic inflammatory state. As shown in Figure 4, TNF‐*α* levels increased from 22.19 ± 3.69 pg/mL in the normal group to 49.61 ± 3.00 pg/mL in the HFD + fructose group. Similarly, IL‐6 levels rose from 20.51 ± 0.71 pg/mL to 44.92 ± 4.06 pg/mL, while CRP levels increased from 0.75 ± 0.11 mg/L to 1.98 ± 0.19 mg/L. These findings demonstrate that HFD + fructose induction significantly enhanced inflammatory responses in the experimental rats. Administration of CPXF reduced all measured inflammatory markers in both treatment groups. In the low‐dose group, TNF‐*α*, IL‐6, and CRP decreased to 36.20 ± 4.89 pg/mL, 34.17 ± 2.63 pg/mL, and 1.39 ± 0.14 mg/L, respectively, compared with the HFD + fructose group. A greater reduction was observed in the high‐dose group, in which TNF‐*α* fell to 29.19 ± 1.32 pg/mL, IL‐6 to 19.57 ± 3.80 pg/mL, and CRP to 0.91 ± 0.17 mg/L.

### 3.8 Effects of CPXF Treatment on Hepatic Injury Markers and Oxidative Stress Parameters

**FIGURE 5:**
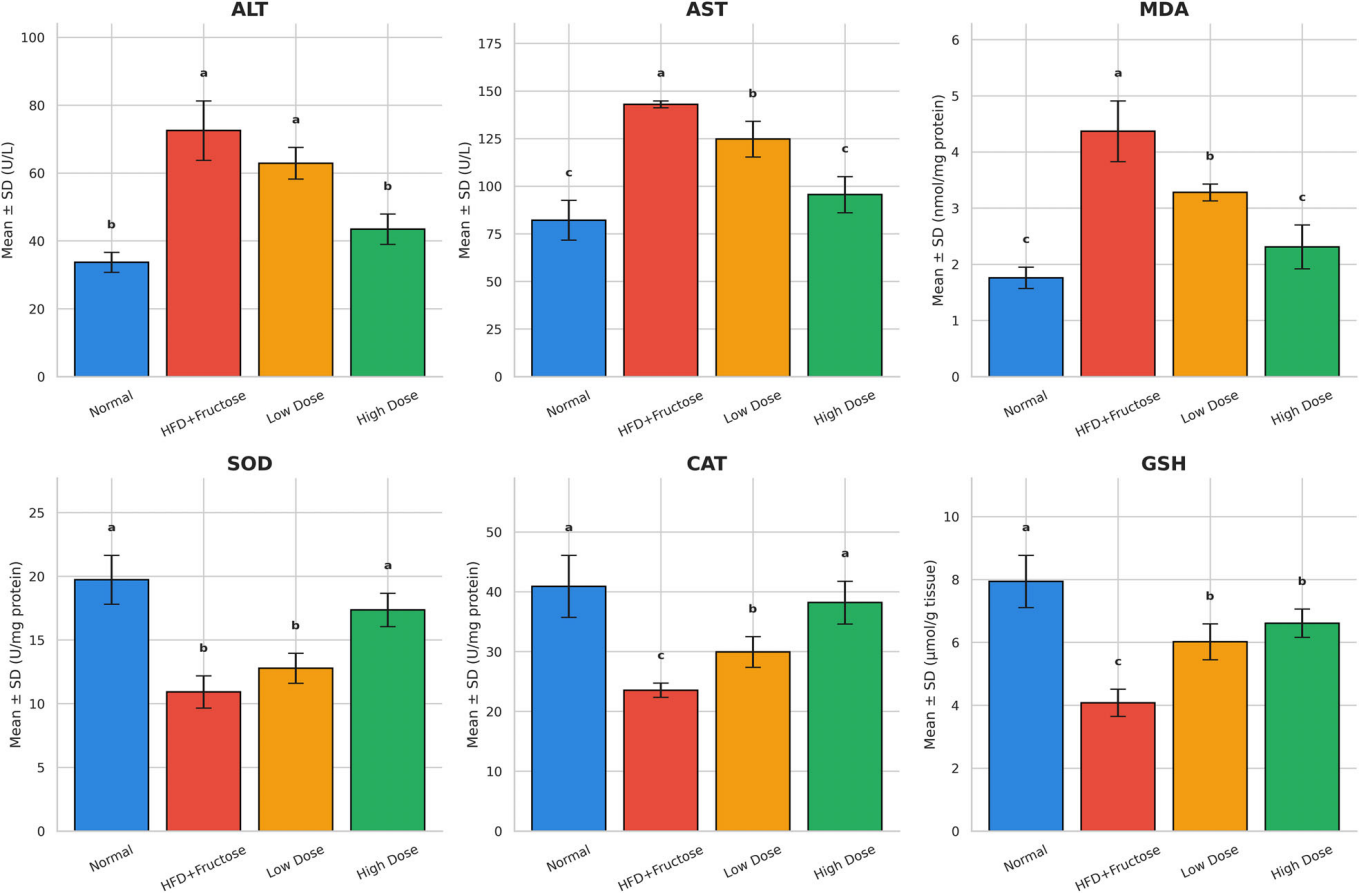
Effect of CPXF treatment on hepatic enzymes (ALT, AST) and oxidative stress parameters (MDA, SOD, CAT, and GSH). Data are expressed as mean ± SD (*n* = 5 per group). Statistical differences were analyzed by one‐way ANOVA followed by Tukey's post hoc test. Different superscript letters or letters above the bars indicate significant differences among groups at *p* < 0.05.

HFD + fructose challenge induced clear evidence of hepatic injury and oxidative imbalance (Figure 5 **)**. Serum ALT and AST were significantly elevated in the HFD + fructose group relative to controls, accompanied by a marked increase in hepatic MDA and a concomitant decline in SOD, CAT, and GSH, indicating enhanced lipid peroxidation and impaired antioxidant defense. Specifically, ALT and AST increased to 72.52 ± 8.76 U/L and 143.00 ± 1.82 U/L, respectively, while MDA rose to 4.37 ± 0.54 nmol/mg protein. In contrast, SOD, CAT, and GSH fell to 10.92 ± 1.26 U/mg protein, 23.55 ± 1.18 U/mg protein, and 4.08 ± 0.43 µmol/g tissue, respectively.

CPXF treatment dose‐dependently reversed these abnormalities. Both doses reduced ALT, AST, and MDA while enhancing SOD, CAT, and GSH, with the high‐dose regimen consistently showing greater efficacy. In the high‐dose group, ALT, AST, and MDA were reduced to 43.46 ± 4.47 U/L, 95.61 ± 9.48 U/L, and 2.31 ± 0.39 nmol/mg protein, respectively, whereas SOD and CAT recovered to 17.36 ± 1.31 and 38.19 ± 3.58 U/mg protein. These changes support a hepatoprotective and antioxidant effect of CPXF in HFD + fructose‐induced metabolic injury.

### 3.9 Effects of CPXF Treatment on Lipid Metabolism‐Related Gene Expression

**FIGURE 6:**
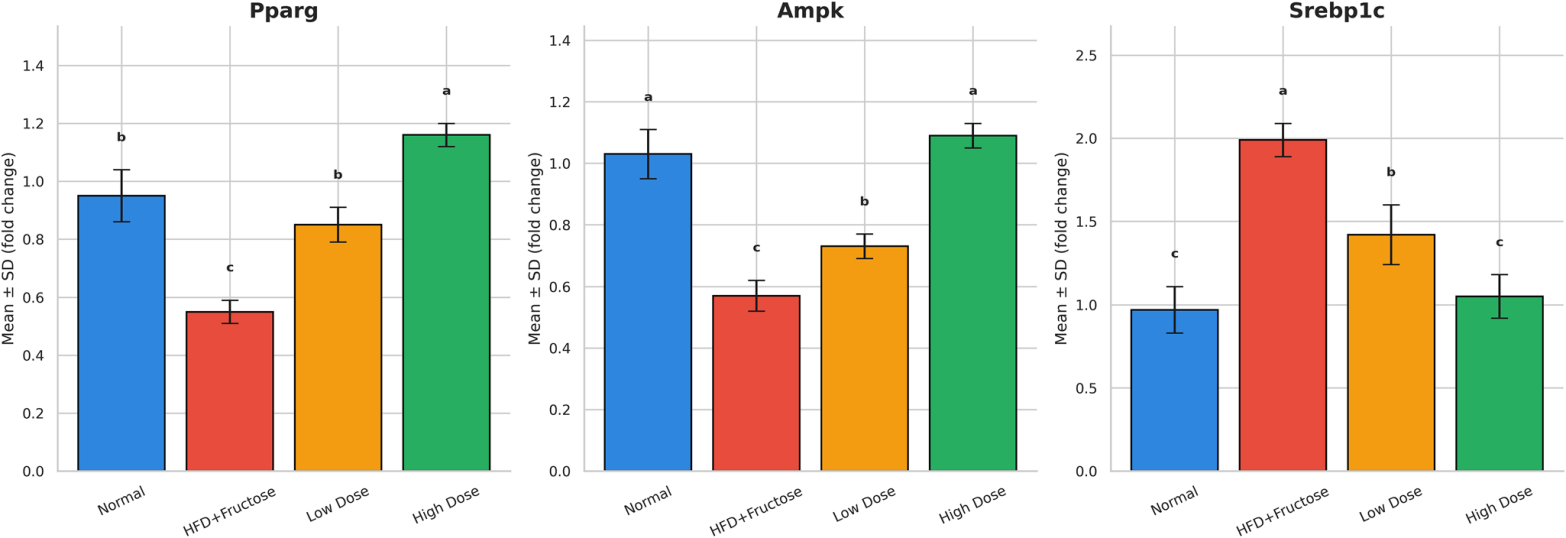
Effect of CPXF treatment on lipid metabolism related gene expression of PPARG, AMPK, and SREBP1c in HFD‐induced rats.

HFD + fructose feeding significantly altered the hepatic expression of genes involved in lipid metabolism regulation (Figure 6 **)**. Compared with the normal group, the HFD + fructose group showed marked downregulation of Pparg and Ampk, decreasing from 0.95 ± 0.09 to 0.55 ± 0.04 fold and from 1.03 ± 0.08 to 0.57 ± 0.05 fold, respectively. In contrast, Srebp1c expression was significantly upregulated, increasing from 0.97 ± 0.14 to 1.99 ± 0.10 fold. These findings indicate that HFD + fructose disrupted hepatic lipid homeostasis by suppressing genes associated with metabolic regulation and energy sensing while enhancing the expression of a key lipogenic transcription factor. CPXF treatment significantly reversed these transcriptional alterations in a dose‐dependent manner. In the low‐dose group, Pparg and Ampk expression increased to 0.85 ± 0.06 and 0.73 ± 0.04 fold, respectively, whereas Srebp1c decreased to 1.42 ± 0.18 fold. A more pronounced effect was observed in the high‐dose group, where Pparg and Ampk increased to 1.16 ± 0.04 and 1.09 ± 0.04 fold, respectively, while Srebp1c declined to 1.05 ± 0.13 fold.

## 4 Discussion

**FIGURE 7:**
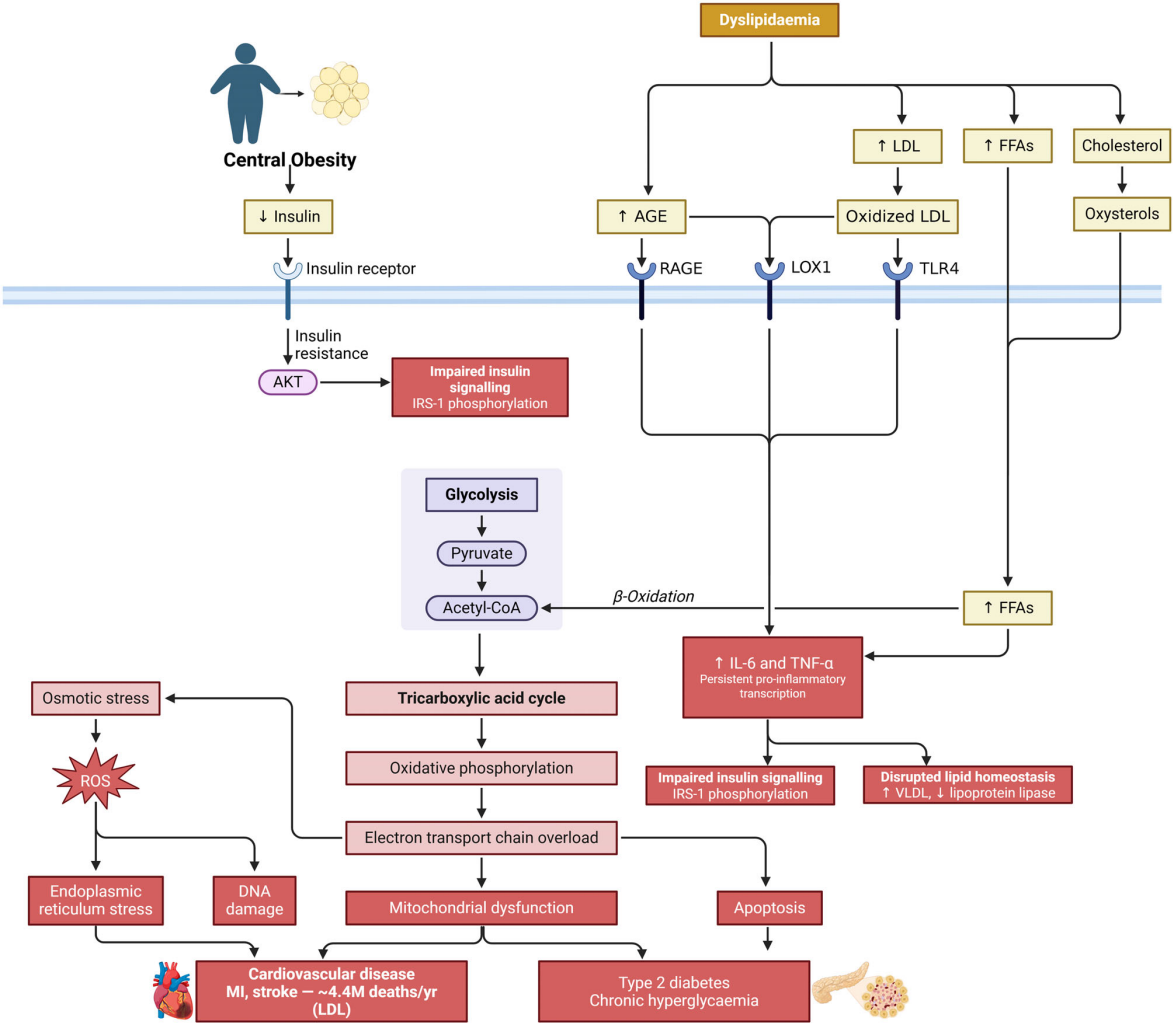
Integrated pathophysiological framework linking central obesity, dyslipidemia, and inflammation to cardiometabolic dysfunction. This figure was created using BioRender.com under a premium license by Fahrul Nurkolis.

The present study shows that CPXF is enriched in xanthophyll carotenoids, particularly BCX, violaxanthin, and lutein, and that this fraction exerts broad protective effects against HFD + fructose‐induced cardiometabolic dysfunction (Figure 7). The HFD combined with fructose supplementation is a well‐validated rodent model of CMS, producing visceral obesity, insulin resistance, dyslipidemia, and chronic inflammation through hepatic lipid overload and fructose‐driven de novo lipogenesis, and successful induction was confirmed by the marked divergence of metabolic parameters from control values. LC–MS/MS profiling revealed that CPXF is dominated by BCX, violaxanthin, and lutein, consistent with established citrus peel phytochemistry [23, 24]. BCX is uniquely bioavailable owing to its intact *β*‐ionone ring, enabling dual metabolism to vitamin A and bioactive apocarotenoids, while the co‐presence of lutein, zeaxanthin, and epoxy‐xanthophylls provides a structurally diverse antioxidant ensemble suited to the multifactorial pathophysiology of CMS [25, 26].

Network pharmacology identified a substantial overlap between CPXF targets and CMS‐associated proteins, with hub nodes centered on PPARG, PRKAA1, SREBF1, and MMP9 a topology mechanistically coherent with CMS, as AMPK–PPAR‐γ governs energy sensing and lipid homeostasis [27]. SREBP‐1c drives hepatic lipogenesis, and MMP9 promotes visceral fibrosis and endothelial dysfunction [28, 29]. Molecular docking further showed that multiple CPXF carotenoids engaged these targets with binding affinities exceeding simvastatin and metformin, consistent with evidence that carotenoid polyene scaffolds interact productively with hydrophobic enzyme pockets, and provide a rational molecular basis for the in vivo outcomes [30].

CPXF produced dose‐dependent improvements in glycemic control and lipid profile, mechanistically anchored in reciprocal hepatic transcriptional shifts: upregulation of *Ampk* and *Pparg* alongside substantial suppression of *Srebp1c*. AMPK activation phosphorylates ACC, reducing malonyl‐CoA and disinhibiting CPT‐1‐mediated fatty acid oxidation while curtailing lipogenesis [31, 32]. PPAR‐γ upregulation promotes ectopic lipid redistribution, possibly reflecting partial agonist activity suggested by the high docking affinity of violaxanthin and zeaxanthin for this receptor [33]. These findings are consistent with meta‐analytic evidence that lutein and zeaxanthin reduce dyslipidemic parameters and prior observations linking BCX to fat oxidation gene upregulation, extending both to a multicomponent citrus fraction acting synergistically across glycemic and lipid axes [14, 34].

CPXF substantially attenuated circulating TNF‐*α*, IL‐6, and CRP, confirming suppression of the inflammatory state that perpetuates insulin resistance in CMS [35]. The magnitude of reduction is comparable to a 6‐month RCT of lutein, zeaxanthin, and meso‐zeaxanthin in humans that achieved significant decreases in IL‐1*β*, TNF‐*α*, and oxidized LDL [36]. Mechanistically, xanthophylls suppress inflammation by blocking I*κB*‐*α* phosphorylation and NF‐*κB*‐driven transcription of pro‐inflammatory mediators [37]. Another studies show that activating Nrf2/Keap1 to upregulate HO‐1 and NQO1, a dual mechanism clinically corroborated by the inverse correlation between plasma lutein plus zeaxanthin and IL‐6 in coronary artery disease patients [38].

Hepatocellular injury markers, lipid peroxidation, antioxidant enzyme activities, and organ weights were all markedly ameliorated by CPXF in a dose‐dependent manner. The hepatoprotective effect reflects membrane‐incorporated xanthophylls interrupting lipid peroxidation chain reactions alongside Nrf2/ARE‐driven induction of SOD, CAT, and glutathione peroxidase. BCX also additionally protects hepatic lipid metabolism via SIRT1/FOXO1 deacetylase signaling [39]. Visceral fat reduction, driven by AMPK‐mediated lipolysis and suppressed adipogenesis, creates a positive feedback loop whereby decreased adipose mass further attenuates adipokine‐driven inflammation, reinforcing the cytokine improvements observed [40].

Collectively, CPXF exerts coordinated, pleiotropic activity across the lipid–glucose–inflammation axis by simultaneously engaging AMPK activation, SREBP‐1c suppression, PPAR‐γ modulation, NF‐*κB* inhibition, Nrf2 induction, and MMP9 attenuation. The consistent dose–response relationship and the breadth of correction across glycemic, lipid, inflammatory, oxidative, and transcriptional domains suggest synergistic interactions among CPXF constituents rather than purely additive effects. A limitation of the present study is the absence of pharmacological positive‐control groups, such as metformin or simvastatin, during the in vivo experiment. The primary objective of this study was to evaluate the intrinsic therapeutic efficacy of CPXF relative to untreated CMS animals, whereas simvastatin and metformin were used solely as reference compounds in the molecular docking analysis. Future studies will directly compare CPXF with established pharmacological therapies to better define its relative therapeutic efficacy and translational potential. Future investigations should include comprehensive safety assessments, including renal biomarkers (urea and creatinine), together with direct comparisons against standard pharmacological therapies such as metformin and simvastatin to further establish the translational value of CPXF. The efficacious doses are achievable with concentrated citrus peel extracts, a sustainable proposition given that peel constitutes approximately half of discarded fruit mass. Future priorities include molecular dynamics simulation, hepatic histological grading, and a Phase I clinical trial to advance CPXF as a food‐derived, multitarget candidate for CMS management.

## 5 Conclusions

In conclusion, CPXF enriched in BCX, violaxanthin, and lutein demonstrated strong binding affinity against key cardiometabolic targets including AMPK, PPAR‐γ, SREBP‐1c, and MMP9 in silico, surpassing reference drugs simvastatin and metformin. In vivo, CPXF dose‐dependently ameliorated insulin resistance, dyslipidemia, visceral adiposity, hepatocellular injury, and oxidative stress, while suppressing circulating TNF‐*α*, IL‐6, and CRP, and restoring hepatic antioxidant enzyme activities. At the transcriptional level, CPXF upregulated Ampk and Pparg while suppressing Srebp1c, providing a mechanistic basis for the observed metabolic improvements. Collectively, these findings support citrus peel xanthophylls as a promising food‐derived, multitarget candidate for CMS management, warranting further clinical investigation.

## Author Contributions

**Muhammad Reva Aditya**: conceptualization, methodology, formal analysis, investigation, data curation, writing – original draft preparation. **Muhammad Yusuf**: methodology, formal analysis, investigation, data curation, writing – original draft preparation. **Kanandya Kizzandy**: methodology, software, investigation, data curation, visualization. **Rizki Hari Mulia**: software, formal analysis, investigation, writing – original draft preparation. **Agnes Vianne**: investigation. **Athaya Rahmanardi Muhammad**: investigation. **Davina Shafa Aviantiputri**: investigation, visualization. **Adha Fauzi Hendrawan**: validation, writing – review and editing. **Ade Meidian Ambari**: validation. **Dante Saksono Harbuwono**: validation, writing – review and editing. **Antonello Santini**:: conceptualization, resources, writing – review and editing, supervision, funding acquisition. **Fahrul Nurkolis**: conceptualization, resources, writing – original draft preparation, writing – review and editing, supervision, project administration, funding acquisition. All authors have read and agreed to the published version of the manuscript.

## Use of Generative AI and AI‐Assisted Technologies in the Writing Process

We acknowledge the use of AI assistance, specifically ChatGPT (Version 5.5), for language refinement and improving the clarity and conciseness of the manuscript. No AI tools were used for data analysis, interpretation, or generating scientific content. All scientific concepts, results, and conclusions were developed and verified by the authors.

## Funding

The authors have nothing to report.

## Ethical Statement

The study protocol was prospectively registered and received ethical approval from the Ethics Committee of the Faculty of Medicine, Brawijaya University (Approval No. 201/EC/KEPK/05/2025).

## Conflicts of Interest

The authors declare no conflicts of interest.

## Institutional Review Board Statement

The study protocol was prospectively registered and received ethical approval from the Ethics Committee of the Faculty of Medicine, Brawijaya University (Approval No. 201/EC/KEPK/05/2025).

## Data Availability

The original contributions presented in this study are included in the article. The datasets used and/or analyzed during the current study are available from the corresponding authors on reasonable request. Further inquiries can be directed to the corresponding authors.
